# Supplementary material for: Effects of the COVID-19 Pandemic on the Interest of Google Queries in Cancer Screening and Cancers: A Retrospective Study
Source: Cancers (Basel). 2023 Jan 19;15(3):617. doi: 10.3390/cancers15030617 (PMC9913796; doi:10.3390/cancers15030617)
Supplement: Supplementary file 1 [file cancers-15-00617-s001.zip › cancers-2097396-supplementary.pdf]

Supplementary Figure S1

The sorted raw difference between actual and forecasted RSV for the chosen topics.

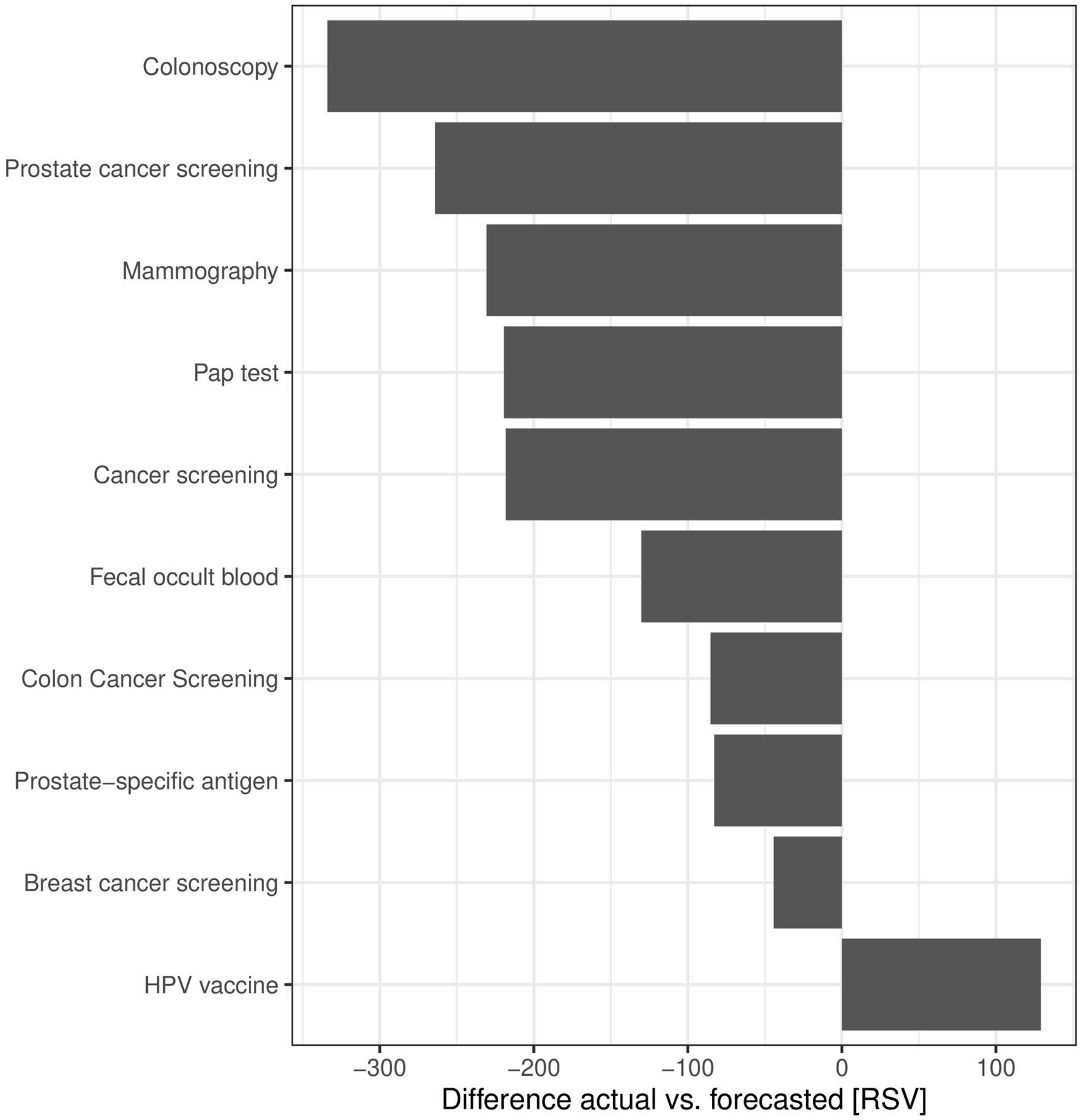

Supplementary Figure S2

The sorted raw difference between actual and forecasted RSV for the chosen topics.

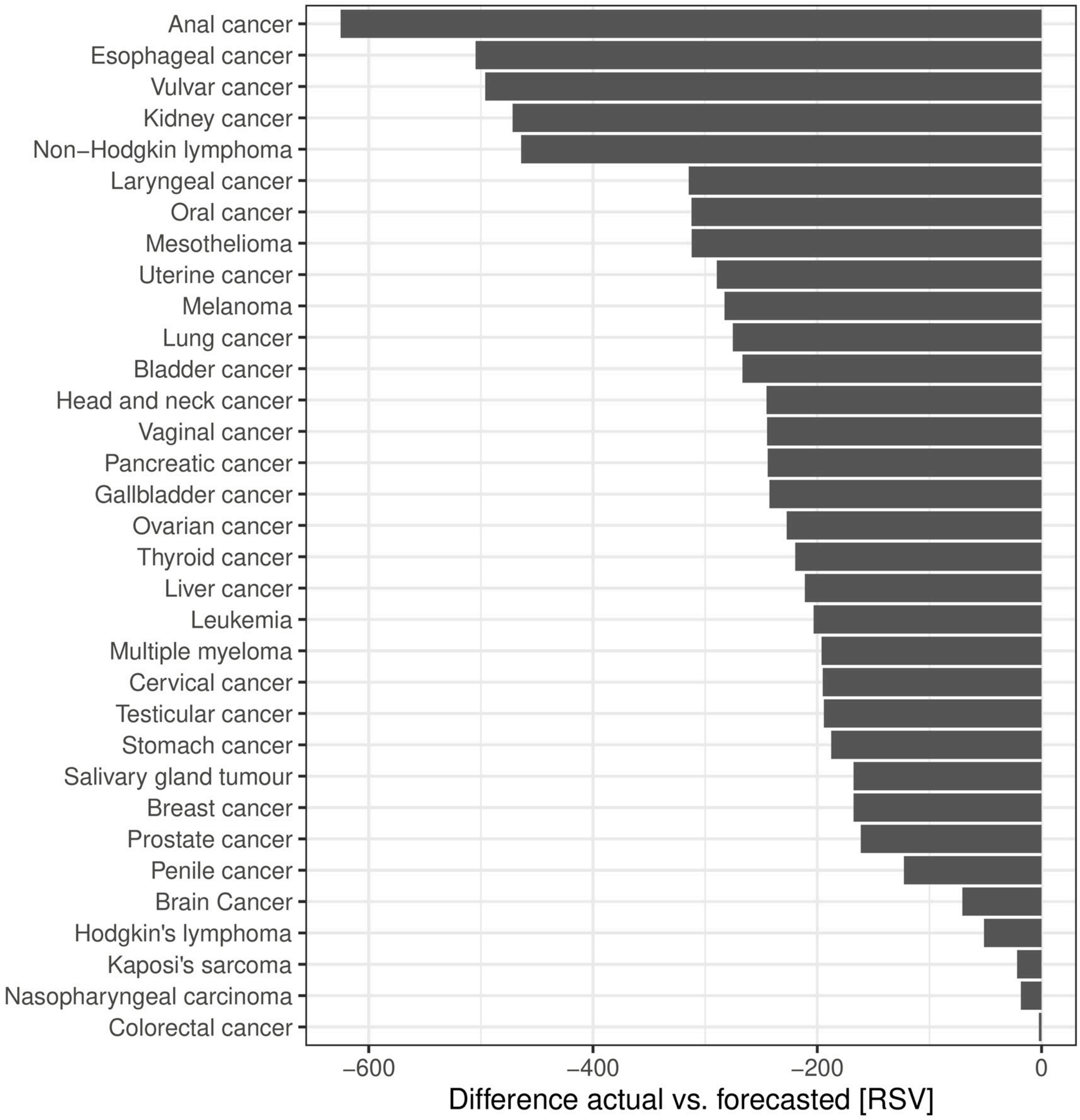

Supplementary Table S1.
Checklist for documentation of Google Trends research. Modified from Nuti et al. [14].

Section/Topic	Checklist item
**Search Variables**	
Access Date	26 February 2022
Time Period	From January 2015 to the end of 2021
Query Category	All query categories were used
Region	Worldwide
Countries with Low Search Volume	Included
**Search Input**	Cancer-related screening topics:<br>"Breast cancer screening", "Cancer screening", "Colon Cancer screening", "Colonoscopy", "Fecal occult blood", "HPV vaccine", "Mammography", "Pap test", "Prostate-specific antigen", "Prostate cancer screening"<br><br>Cancer name topics:<br>"Anal cancer", "Bladder cancer", "Brain Cancer", "Breast cancer", "Cervical cancer", "Colorectal cancer", "Esophageal cancer", "Gallbladder cancer", "Head and neck cancer", "Hodgkin's lymphoma", "Kaposi's sarcoma", "Kidney cancer", "Laryngeal cancer", "Leukemia", "Liver cancer", "Lung cancer", "Melanoma", "Mesothelioma", "Multiple myeloma", "Nasopharyngeal carcinoma", "Non-Hodgkin lymphoma", "Oral cancer", "Ovarian cancer", "Pancreatic cancer", "Penile cancer", "Prostate cancer", "Salivary gland tumour", "Stomach cancer", "Testicular cancer", "Thyroid cancer", "Uterine cancer", "Vaginal cancer", "Vulvar cancer"
**Rationale for Search Strategy**	
For Search Input	The searched topics for both cancer names and cancer-related screenings. We only included terms that could be matched as topics.
For Setting Chosen	We chose all categories to not limit the output.

Supplementary Table S2

Sensitivity analysis after exclusion of unexpected peaks of interest and data for March and April 2020.

If the difference became insignificant ($p > 0.05$) but was significant in the main analysis (Tables 1 and 2), the p-value is highlighted in red, and the reverse is highlighted in green.

A) Cancer-screening-related topics and HPV vaccine

Name of screening-related topic	Before pandemic (RSV)	Forecasted interest (RSV)	Actual interest (RSV)	Difference: actual vs. forecast (RSV)	Prepandemic vs. during pandemic* (p-value)	Forecasted vs. actual** (p-value)
Breast cancer screening	50.0 (47.0-53.2)	56.2 (52.0-60.0)	57.5 (52.5-60.0)	-44.3	**0.019**	0.97
Cancer screening	67.0 (54.8-73.2)	87.7 (84.1-91.2)	79.0 (76.2-91.2)	-218.2	**< 0.001**	**< 0.01**
Colon Cancer Screening	43.0 (38.0-55.2)	58.3 (58.3-58.3)	55.0 (45.0-58.3)	-85.3	**0.025**	0.16
Colonoscopy	65.0 (61.0-74.5)	93.2 (88.5-96.9)	85.0 (76.0-96.9)	-334.0	**< 0.001**	**< 0.001**
Fecal occult blood	74.0 (61.0-79.2)	82.5 (80.5-86.4)	81.0 (75.0-86.4)	-130.2	**< 0.01**	0.24
HPV vaccine	58.5 (49.8-68.2)	68.6 (66.3-75.5)	75.0 (69.0-75.5)	129.2	**< 0.001**	**0.016**
Mammography	54.0 (48.8-60.0)	71.4 (67.2-74.6)	64.0 (59.5-74.6)	-230.7	**< 0.001**	**< 0.01**
Pap test	82.0 (77.8-88.5)	92.8 (90.1-93.4)	87.0 (84.0-93.4)	-219.4	0.11	**< 0.01**
Prostate-specific antigen	67.0 (47.0-77.2)	86.0 (83.7-88.4)	86.5 (83.0-88.4)	-82.9	**< 0.001**	0.90
Prostate cancer screening	63.0 (56.0-74.0)	77.8 (73.5-81.9)	71.5 (62.5-81.9)	-264.1	0.08	**< 0.01**

RSV—Relative Search Volume, * unpaired comparison of RSV before the COVID-19 pandemic vs. the actual RSV during the pandemic, **unpaired comparison of RSV of the forecasted trend vs. the actual RSV during the pandemic.

B) Cancer-related topics

Name of malignancy	Before pandemic (RSV)	Forecasted interest (RSV)	Actual interest (RSV)	Difference: actual vs. forecast (RSV)	Prepandemic vs. during pandemic* (p-value)	Forecasted vs. actual** (p-value)
Anal cancer	42.0 (32.0-48.2)	71.6 (67.4-76.2)	46.0 (41.8-76.2)	-625.1	0.06	**< 0.001**
Bladder cancer	81.0 (76.0-88.0)	92.9 (91.4-95.1)	84.5 (80.0-95.1)	-266.7	0.24	**< 0.001**
Brain Cancer	4.0 (2.0-7.0)	4.5 (4.5-4.5)	1.0 (1.0-4.5)	-70.6	**< 0.001**	**< 0.001**
Breast cancer	47.0 (45.0-50.2)	47.5 (46.3-50.1)	42.5 (41.0-50.1)	-167.7	**< 0.001**	**< 0.001**
Cervical cancer	75.0 (73.0-78.0)	75.0 (73.7-77.3)	68.0 (64.5-77.3)	-195.1	**< 0.001**	**< 0.001**
Colorectal cancer	41.0 (40.0-44.0)	44.5 (42.6-45.1)	43.0 (39.0-45.1)	-2.4	0.32	0.28
Esophageal cancer	77.0 (71.0-84.0)	101.5 (96.9-105.1)	81.0 (78.2-105.1)	-504.7	0.09	**< 0.001**
Gallbladder cancer	75.0 (69.0-81.2)	78.8 (78.8-78.8)	72.5 (66.8-78.8)	-242.7	**0.031**	**< 0.001**
Head and neck cancer	71.0 (67.8-75.2)	80.9 (78.8-82.5)	72.0 (68.5-82.5)	-245.3	0.99	**< 0.001**
Hodgkin's lymphoma	40.0 (37.0-42.0)	43.6 (41.5-45.9)	41.0 (37.2-45.9)	-51.3	0.52	**< 0.001**
Kaposi's sarcoma	57.0 (52.8-59.2)	49.6 (47.8-51.3)	49.0 (46.2-51.3)	-21.8	**< 0.001**	0.46
Kidney cancer	84.0 (78.8-89.2)	98.7 (96.7-100.7)	81.5 (77.2-100.7)	-471.7	0.11	**< 0.001**
Laryngeal cancer	74.5 (69.8-81.0)	85.2 (85.2-85.2)	72.0 (69.2-85.2)	-314.6	0.17	**< 0.001**
Leukemia	58.0 (56.0-61.0)	63.0 (60.5-63.8)	54.0 (52.2-63.8)	-203.3	**< 0.001**	**< 0.001**
Liver cancer	69.5 (66.0-83.2)	66.8 (66.8-66.8)	60.0 (58.0-66.8)	-211.1	**< 0.001**	**< 0.001**
Lung cancer	81.0 (76.8-84.2)	82.6 (80.8-87.4)	72.0 (69.2-87.4)	-275.3	**< 0.001**	**< 0.001**
Melanoma	77.0 (70.0-81.0)	79.8 (77.8-85.3)	72.0 (65.5-85.3)	-282.7	**< 0.01**	**< 0.001**
Mesothelioma	47.0 (39.0-54.5)	70.5 (66.5-74.6)	55.0 (52.0-74.6)	-312.0	**< 0.01**	**< 0.001**
Multiple myeloma	67.5 (63.0-72.2)	79.8 (77.7-81.2)	70.0 (68.0-81.2)	-196.2	**0.027**	**< 0.001**
Nasopharyngeal carcinoma	34.5 (31.0-38.0)	35.0 (34.4-36.3)	36.5 (35.0-36.3)	-18.4	0.25	0.37

Name of malignancy	Before pandemic (RSV)	Forecasted interest (RSV)	Actual interest (RSV)	Difference: actual vs. forecast (RSV)	Prepandemic vs. during pandemic* (p-value)	Forecasted vs. actual** (p-value)
Non-Hodgkin lymphoma	66.0 (57.8-73.2)	80.2 (79.6-80.7)	61.0 (55.2-80.7)	-464.1	0.08	**< 0.001**
Oral cancer	61.0 (58.0-65.0)	68.2 (66.4-69.3)	56.0 (54.0-69.3)	-312.2	**< 0.001**	**< 0.001**
Ovarian cancer	76.5 (73.0-79.0)	75.0 (75.0-75.0)	66.5 (64.0-75.0)	-227.3	**< 0.001**	**< 0.001**
Pancreatic cancer	55.0 (48.8-61.0)	66.7 (66.7-66.7)	57.0 (56.2-66.7)	-244.2	0.10	**< 0.001**
Penile cancer	81.0 (77.0-86.2)	65.9 (65.9-65.9)	61.0 (59.0-65.9)	-122.7	**< 0.001**	**< 0.001**
Prostate cancer	75.5 (68.8-83.0)	85.6 (84.6-90.0)	82.0 (80.2-90.0)	-161.2	**< 0.01**	**< 0.01**
Salivary gland tumour	81.0 (74.0-88.0)	85.1 (79.4-88.5)	79.5 (71.5-88.5)	-167.7	0.62	0.12
Stomach cancer	84.0 (82.0-89.0)	89.2 (89.2-89.2)	84.0 (82.2-89.2)	-187.6	0.30	**< 0.001**
Testicular cancer	72.5 (69.0-79.2)	75.1 (75.1-75.1)	67.5 (66.0-75.1)	-194.1	**< 0.001**	**< 0.001**
Thyroid cancer	87.0 (84.0-90.2)	89.5 (86.0-91.0)	82.0 (76.8-91.0)	-219.7	**< 0.01**	**< 0.01**
Uterine cancer	84.0 (80.0-87.0)	85.1 (83.0-86.0)	73.5 (71.0-86.0)	-289.6	**< 0.001**	**< 0.001**
Vaginal cancer	75.5 (70.8-82.2)	84.9 (84.9-84.9)	74.5 (71.0-84.9)	-244.8	0.66	**< 0.001**
Vulvar cancer	58.0 (52.8-62.5)	94.3 (91.4-97.4)	75.5 (66.8-97.4)	-496.1	**< 0.001**	**< 0.001**

RSV—Relative Search Volume, * unpaired comparison of RSV before the COVID-19 pandemic vs. the actual RSV during the pandemic, **unpaired comparison of RSV of the forecasted trend vs. the actual RSV during the pandemic.
